# Short term outcomes of total arterial coronary revascularization in patients above 65 years: a propensity score analysis

**DOI:** 10.1186/1749-8090-5-25

**Published:** 2010-04-18

**Authors:** Wael Hassanein, Yasser Y Hegazy, Alexander Albert, Ina C Ennker, Ulrich Rosendahl, Stefan Bauer, Juergen Ennker

**Affiliations:** 1 Cardiac Surgery Department, Heart Institute Lahr/Baden - Germany; 2 Clinic of Cardiovascular Surgery, Duesseldorf University Hospital - Germany

## Abstract

**Background:**

Despite the advantages of bilateral mammary coronary revascularization, many surgeons are still restricting this technique to the young patients. The objective of this study is to demonstrate the safety and potential advantages of bilateral mammary coronary revascularization in patients older than 65 years.

**Methods:**

Group I included 415 patients older than 65 years with exclusively bilateral mammary revascularization. Using a propensity score we selected 389 patients (group II) in whom coronary bypass operations were performed using the left internal mammary artery and the great saphenous vein.

**Results:**

The incidence of postoperative stroke was higher in group II (1.5% vs. 0%, P = 0.0111). The amount of postoperative blood loss was higher in group I (908 ± 757 ml vs. 800 ± 713 ml, P = 0.0405). There were no other postoperative differences between both groups.

**Conclusion:**

Bilateral internal mammary artery revascularization can be safely performed in patients older than 65 years. T-graft configuration without aortic anastomosis is particularly beneficial in this age group since it avoids aortic manipulation, which is an important risk factor for postoperative stroke.

## Background

The world's population has been aging rapidly over the past 50 years. Currently 11% of the world's population and 22% of the developed regions' population are older than 60 years and these ratios are expected to increase [1]. This steady increase in the number of the elderly will be ultimately reflected on the demographic aspects of the patients subjected to coronary bypass operations. With increasing life expectancy of the patients, cardiac surgeons are urged to give more attention to the long-term results of their operations.

The internal mammary artery has been considered as the optimal conduit because of its superior patency rate and freedom from arteriosclerosis [2]. The long term advantages of bilateral internal mammary artery grafting in comparison with left internal mammary with vein grafts are well documented [3-5]. The mid-term results of bilateral internal mammary revascularization were also reported in the elderly [6,7].

Despite the accumulating evidences supporting the advantages of bilateral mammary revascularization, most of the surgeons are still reluctant to adopt this technique especially in the elderly patients. This indicates that the evidence supporting the short term safety of bilateral mammary revascularization is not as strong as that supporting its long term advantages.

The objective of this study is to demonstrate the feasibility, safety and potential advantages of exclusive bilateral mammary revascularization in the patients older than 65 years.

## Methods

From January 1996 till December 2008 we performed 11,254 isolated elective coronary bypass operations including 1297 total arterial revascularization using exclusively bilateral internal mammary arteries. The decision to perform total arterial revascularization was taken on individual basis by the surgeon after discussing the different options with the patient.

Among the patients operated upon with total arterial revascularization, there were 415 patients older than 65 years (group I). Patients with previous cardiac operations and those with ejection fraction less than 30% were not included in the search. Using a propensity score [8] we selected 389 patients from our database (group II) in whom isolated elective coronary bypass operations were performed using exclusively the left internal mammary artery and the great saphenous vein.

All patients signed informed consent for the operation and data collection.

### Operative management

All operations were performed through conventional sternotomy. All internal mammary arteries were harvested skeletonized. Papaverine was sprayed on, but not injected inside the mammary arteries.

Intravenous heparin (300 IU/kg) was given to maintain activated clotting time above 480 seconds in both on-pump and off-pump cases. The target cardiopulmonary bypass flow was maintained between 90%-120% of the calculated value (2.5 l/m^2^). The target pressure was 60 mmHg, and higher for patients with known carotid stenosis (60-80 mmHg), maintained with noradrenalin if necessary.

The cardiopulmonary bypass was conducted under systemic normothermia and antegrade cold hyperkalemic blood cardioplegia. Bypass grafting was performed under single aortic cross clamp.

Off-pump cases were performed using suction stabilisers such as Octopus™ (Medtronic Inc., Minneapolis, MN, USA) or the Axius Vacuum Stabilizer System™ (Guidant Corporation, Santa Clara, CA, USA). In most of cases heart positioners were used: Starfish Heart Positioner ™ (Medtronic Inc., Minneapolis, MN, USA) and Xpose Access Devise ™ (Guidant Corporation, Santa Clara, CA, USA). Intracoronary shunts were used during performing the anastomses in all off-pump cases. A blower-mister was used to help visibility.

In group I, a T-graft configuration was used in all cases with the left internal mammary anastomosed to the LAD and the right internal mammary to all other coronary arteries in a sequential manner. In group II, the left internal mammary was anastomosed to the LAD and the vein graft to the other coronary vessels. The vein grafts were anastomosed proximally to the aorta in 265 patients and as a T-graft to the internal mammary artery in 124 patients operated upon using the aorta no-touch technique.

### Definition of terms

Patients were considered to have preoperative renal insufficiency when the preoperative creatinine clearance was less than 60 ml/min or serum creatinine was higher than 1.5 mg/dL or when there was a history of hemodialysis. Preoperative liver insufficiency was considered based on the diagnosis made by the treating physician.

Postoperative outcomes are those events occurring within 30 days of the operation. Deep sternal wound infection was considered, following the guidelines of the Centres for Disease Control and Prevention [9]. Postoperative myocardial infarction was defined by the elevation of creatine phosphokinase-MB fraction more than 50 U/L with the appearance of new Q waves in the ECG. Carotid stenosis was defined as occlusion or more than 50% stenosis of at least one common carotid or internal carotid artery. Postoperative stroke was defined as new focal or global neurological deficit, lasting more than 24 hours, diagnosed by a neurologist and/or confirmed by a brain CT scan.

### Statistical analysis

Data were collected in all patients using standardized protocols of the German Society of Thoracic and Cardiovascular Surgery and Intensive Care Medicine [10,11]. A technical assistant for data collection and medical documentation controlled the data collection and tested its reliability. Data were extracted using dedicated project oriented data warehouse (data-mart) where it got transformed, consolidated, and several plausibility checks were performed. All statistics were obtained by JMP 5.1 software (SAS Institute, Inc, Cary, NC)

A propensity score was used to select the patients of group II. The details of propensity score analysis has been published elsewhere [8]. We used propensity score analysis to estimate the probability that a patient might be assigned exclusively bilateral internal mammary revascularization rather than revascularization using exclusively the left internal mammary artery and the great saphenous vein. Confounding preoperative factors, demographic and operative variables, that might have been in favour of one technique to the other or that could affect the results, were listed and then entered into a logistic regression model to obtain a propensity score for each patient. We matched at least one patient from group I with one patient from group II with similar propensity score value (a difference of propensity score for a matching up to 0.05 was allowed).

Variables included in the propensity score model:

• Age

• Female gender

• Chronic Obstructive Pulmonary Disease (COPD)

• EuroSCORE

• Ejection Fraction (EF)

• Peripheral arterial vascular disease (PAD)

• Renal insufficiency

• Off-pump (OPCAB)

The goodness of model was evaluated using the Hosmer and Lemeshow goodness-of-fit statistic and residual analysis. The propensity score model C-statistics (area under the receiver operating characteristic curve) was 0.82 indicating excellent matching between the two groups.

Data were expressed as mean values ± Standard deviation (SD) as well as 25, 50 and 75 percentile. Continuous variables were evaluated by unpaired Student's t test or Pearson test. For comparison of categorical variables X^2 ^test and Fisher exact test were used, together with odds ratio and 95% confidence interval (CI 95%). P values less than 0.05 were considered statistically significant.

## Results

There were no important differences between the two groups regarding the preoperative characteristics (Tables 1 and 2).

**Table 1:** Preoperative categorical variables (ACVB 389 - TAR 415)

	ACVB		TAR		P	Odds ratio	CI 95%	
				
	n	%	n	%			Lower	Upper
COPD	64	16.4	84	20.24	0.166	1.288	0.9008	1.850

DM	111	28.5	131	31.5	0.349	1.155	0.854	1.564

Females	105	26.9	110	26.51	0.876	0.975	0.713	1.333

Renal insufficiency	48	12.34	49	11.8	0.816	0.951	0.621	1.455

Liver insufficiency	19	4.88	18	4.34	0.711	0.882	0.453	1.714

Atrial fibrillation	16	4.11	24	5.78	0.2745	1.430	0.754	2.785

PAD	45	11.57	40	9.64	0.374	0.815	0.518	1.279

Hypertension	315	80.98	368	88.95	**0.0022**	1.839	**1.243**	**2.745**

Pulm. Hypertension	6	1.54	4	0.96	0.4586	0.621	0.157	2.191

Carotid stenosis	72	18.51	70	16.8	0.541	0.893	0.621	1.283

Angina Pectoris	111	28.2	129	31.1	0.578	1.224	0.685	2.189

ACVB = Aorto-Coronary Venous Bypass, TAR = Total Arterial Revascularization, COPD = Chronic Obstructive Pulmonary Disease, DM = Diabetes Mellitus, PAD = Peripheral Arterial Disease

**Table 2:** Preoperative continuous variables (ACVB 389 vs. TAR 415)

		Min.	25%	50%	75%	Max	Mean	Std. Dev.	P
Age (years)	ACVB	65.08	68	70.92	75.83	88	72.065	4.866	0.532
		
	TAR	65.08	67.92	71.42	75.25	88.08	71.859	4.472	

BMI (kg/m^2^)	ACVB	18.22	24.87	27.04	29.40	43.25	27.365	3.645	**0.0155**
		
	TAR	17.67	25.46	27.89	30.1	41.14	27.97	3.501	

EF (%)	ACVB	30	50	61	70	88	59.52	13.25	0.138
		
	TAR	30	52	65	70	91	60.91	13.15	

EuroSCORE	ACVB	2	3	5	6	12	5.020	2.223	0.1911
		
	TAR	2	3	4	6	12	4.816	2.184	

Hb (g/dl)	ACVB	8	12.5	13.6	14.6	18.1	13.45	1.555	0.177
		
	TAR	8.8	12.6	13.7	14.7	17.5	13.60	1.552	

S. Urea (mg/dl)	ACVB	17	33	39	48	341	42.86	22.23	0.401
		
	TAR	13	32	39	47	133	41.74	14.29	

BMI = Body Mass Index, EF = Ejection Fraction, Hb = Haemoglobin, S Urea = Serum Urea

The number of peripheral anastomoses ranged from 2 to 6 in both groups with a mean of 3.14 ± 0.86 in group I vs. 3.03 ± 0.8 in group II, P = 0.063. OPCAB was performed in 185 patients (44.6%) in group I vs. 173 patients (44.4%) in group II (P = 0.976). Among the OPCAB subgroup of group II, there were 124 patients operated upon using the aorta no-touch technique. Partial aortic clamping was performed in the other 49 patients. The mean operative time was 197.6 ± 42.4 minutes in group I vs. 191 ± 44.3 minutes in group II (P = 0.033).

The incidence of postoperative stroke was significantly higher in group II (6 patients (1.5%) vs. no patients (0%), P = 0.0111). In group II, 4 cases of stroke occurred in patients operated upon using the cardiopulmonary bypass. The other 2 cases occurred in the OPCAB subgroup with partial clamping of the aorta.

The difference in stroke between the two OPCAB subgroups fell short of the statistically significant level (P = 0.69). There were no significant differences between the both OPCAB subgroups regarding the postoperative results.

The amount of postoperative blood loss was higher in group I (908 ± 757 ml vs. 800 ± 713 ml, P = 0.0405). There were no other postoperative differences between both groups (Tables 3 and 4).

**Table 3:** Postoperative categorical variables (ACVB 389 vs. TAR 415)

	ACVB		TAR		P	Odds ratio	CI 95%	
				
	n	%	n	%			Lower	Upper
DSWI	7	1.8	10	2.4	0.54	0.74	0.26	1.951

Arrhythmia	172	44.2	175	42.2	0.5581	0.919	0.695	1.216

Reintubation	16	4.1	12	2.8	0.3451	0.694	0.317	1.479

Stroke	6	1.5	0	0	**0.0111**			

Infarction	12	3.1	7	1.6	0.1922	0.539	0.198	1.354

Rethoracotomy	6	1.5	5	1.2	0.6805	0.778	0.222	2.604

30 days Mortality	4	1	7	1.7	0.4219	1.651	0.494	6.343

DSWI = Deep Sternal Wound Infection

**Table 4:** Postoperative continuous variables (ACVB 389 vs. TAR 415)

		Min.	25%	50%	75%	Max.	Mean	St.D.	P
ICU Stay (days)	ACVB	1	2	3	5.75	35	4.63	3.93	0.3951
		
	TAR	1	2	3	6	40	4.87	3.43	

Blood loss (ml)	ACVB	0	400	625	1025	8300	800.01	713.24	**0.0405**
		
	TAR	0	475	750	1150	7880	907.88	756.79	

Pd Hb (g/dl)	ACVB	7.7	10.4	11.4	12.4	14.9	11.40	1.318	0.5783
		
	TAR	8.1	10.5	11.3	12.2	14.8	11.35	1.24	

Max LC(1000/ul)	ACVB	3.2	10.3	12.6	14.9	59.3	13.32	5.09	0.7021
		
	TAR	5.7	10.1	12.1	14.7	82.7	13.17	5.58	

S Urea (mg/dl)	ACVB	17	33	39	48	341	42.86	22.23	0.4017
		
	TAR	13	32	39	47	133	41.74	14.29	

Pd = Predischarge, LC = Leucocytic count, S Urea = Serum Urea (highest measurement)

## Discussion

The long term advantages of bilateral internal mammary artery grafting in comparison with left internal mammary with vein grafts are well documented [3-5]. Recently, Mohammadi et al [12] conducted a study aiming to find an age-cut-off for the loss of benefit from bilateral internal mammary artery grafting. They studied more than 10,000 patients and concluded that the additional survival benefit of using a second internal mammary artery decreases gradually with age, and is lost after 60 years of age. Concerns regarding the technical aspects of this work have already been published [13]. As a matter of fact, old age is not known to be a protective factor against occlusion of vein grafts. Loss of long term benefit of bilateral mammary can always be statistically demonstrated if only few patients survive long enough to reach the time where venous grafts are occluded while arterial grafts are still patent. Prospectively speaking, the surgeon can never know how long his next patient is going to live after the operation. We believe that setting a concrete cut-off age for applying total arterial revascularization is not the best practice. However, we chose to study the patients older than 65 years because this is the age at which it was recommended not to perform bilateral mammary revascularization [12].

An important factor negatively influencing the decision to perform total arterial revascularization is the lack of general acceptance about the optimal strategy of arterial bypass grafting. In our group of patients with total arterial revascularization we included only patients with exclusively bilateral internal mammary in a T-graft configuration with the left mammary supplying the LAD and the right mammary supplying the other coronary vessels. This strategy has become our standard bypass procedure in all age groups. According to our experience, it is possible in the vast majority of patients to perform total revascularization using this strategy. We developed a simple formula to estimate the required length of the right internal mammary artery preoperatively [14].

In T-graft composite bilateral internal mammary revascularization, the whole heart depends on the left internal mammary for its blood supply. Concerns regarding the inability of the left internal mammary to supply the whole heart are only theoretical. These concerns are not supported by well-designed studies and are not evidence based. On the other hand, important studies showed that total arterial revascularization using a composite graft provided a 2-3 fold increase of reserve blood flow to the coronary vascular bed [15,16].

An important advantage of bilateral mammary revascularization with the T-graft configuration is minimizing the risk of stroke by avoiding performing the proximal anastomosis to the ascending aorta. In our 415 patients there was no single patient with postoperative stroke. Embolic dislodgment of atherosclerotic plaques during surgical aortic manipulations has been recognised as a major cause of stroke [17]. This is particularly important in the elderly patients. Avoiding aortic manipulations results in a minimal incidence of perioperative stroke [18].

An apparent disadvantage of bilateral mammary revascularization is the increase in amount of postoperative blood loss. In our study, the patients of the total arterial group lost about 100 ml blood through the chest drains more than those of the conventional group. This increase in blood loss was also observed in other studies [19]. In the presence of a second mammary bed, more blood loss through the chest drains should be expected. Nevertheless, this increase in chest drainage becomes clinically less relevant if we take in consideration the avoidance of blood loss through the leg wound.

An important concern about bilateral mammary revascularization is the sternal wound complications. Tampoulis et al [20] presented a best evidence topic according to a structured protocol to answer the question, if bilateral mammary coronary bypass increases the risk for mediastinitis. Their results showed that bilateral mammary revascularization carried 2.5 to 5 fold higher incidence for mediastinitis after coronary bypass. Nevertheless, in patients in whom the internal mammary was harvested skeletonized, the risk was significantly lower and almost similar to patients receiving a single internal mammary graft. Harvesting the internal mammary artery together with the fascia, vein, muscle and fat is likely to compromise the blood supply to the sternum impending the sternal healing and exposing the sternum to the risk of early dehiscence and infections. In our study we used skeletonized internal mammary arteries in all the patients and we found no statistically significant difference between our two groups of patients. All 17 DSWI cases (7 in ACVB and 10 TAR) were treated using vacuum-assisted closure.

The decreased incidence of mediastinitis with skeletonised internal mammary artery has no patency cost. Calafiore et al [21] demonstrated that skeletonised and pedicled internal mammary arteries are equal regarding the early and midterm postoperative patency.

In conclusion, total arterial revascularization using exclusively the two internal mammary arteries is safe to perform in the elderly. T-graft configuration without aortic anastomosis is particularly beneficial in this age group since it avoids aortic manipulation, which is an important risk factor for postoperative stroke.

### Limitations

An important limitation of our study is the lack of longer follow up. However, the long term advantages of bilateral internal mammary artery grafting in comparison with left internal mammary with vein grafts are well documented [3-5].

Another limitation is its retrospective nature. To overcome this limitation, we performed the propensity score analysis. Nevertheless, propensity score analysis has its own limitations [8].

## Competing interests

The authors declare that they have no competing interests.

## Authors' contributions

WH wrote the first draft of the manuscript. YYH wrote the "Results" section. AA helped with data collection and retrieval, and performed the statistical analysis. JE approved the final version of the manuscript. All authors revised the manuscript critically.
